# Haplotype data for 23 Y-chromosome markers in a reference sample from Bosnia and Herzegovina

**DOI:** 10.3325/cmj.2013.54.286

**Published:** 2013-06

**Authors:** Lejla Kovačević, Vera Fatur-Cerić, Negra Hadžić, Jasmina Čakar, Dragan Primorac, Damir Marjanović

**Affiliations:** 1 Faculty of Pharmacy, University in Sarajevo, Sarajevo, Bosnia and Herzegovina; 2 Institute for Genetic Engineering and Biotechnology, Sarajevo, Bosnia and Herzegovina; 3 University of Split, School of Medicine and Department of Forensic Science, Split, Croatia; 4 University of Osijek School of Medicine, Osijek, Croatia; 5 Eberly College of Science, Penn State University, University Park, PA, USA; 6 University of New Haven, New Haven, CT, USA; 7 Genos Ltd, Zagreb, Croatia

## Abstract

**Aim:**

To detect polymorphisms of 23 Y-chromosomal short tandem repeat (STR) loci, including 6 new loci, in a reference database of male population of Bosnia and Herzegovina, as well as to assess the importance of increasing the number of Y-STR loci utilized in forensic DNA analysis.

**Methods:**

The reference sample consisted of 100 healthy, unrelated men originating from Bosnia and Herzegovina. Sample collection using buccal swabs was performed in all geographical regions of Bosnia and Herzegovina in the period from 2010 to 2011. DNA samples were typed for 23 Y STR loci, including 6 new loci: DYS576, DYS481, DYS549, DYS533, DYS570, and DYS643, which are included in the new PowerPlex^®^ Y 23 amplification kit.

**Results:**

The absolute frequency of generated haplotypes was calculated and results showed that 98 samples had unique Y 23 haplotypes, and that only two samples shared the same haplotype. The most polymorphic locus was DYS418, with 14 detected alleles and the least polymorphic loci were DYS389I, DYS391, DYS437, and DYS393.

**Conclusion:**

This study showed that by increasing the number of highly polymorphic Y STR markers, to include those tested in our analysis, leads to a reduction of repeating haplotypes, which is very important in the application of forensic DNA analysis.

The highly polymorphic short tandem repeat (STR) loci located on the Y chromosome in the male human genome are widely used for forensic and paternity testing and population genetic studies (1-3). Currently, in response to the requirement for increasing the number of Y-STR markers included in some Y-STR multiplex kits, Promega developed the PowerPlex® Y 23 amplification kit (Promega Corporation, Madison, WI, USA), which we used in this study.

Previously, population studies of the male reference sample of Bosnia and Herzegovina were performed by analyzing 12 Y-chromosomal STR loci incorporated in the PowerPlex® Y 12 amplification kit (Promega Corporation) (4), various numbers of Y-SNP markers (5), as well as autosomal (6,7), and X-STR markers (8). All obtained results were included in the reference database of Bosnia and Herzegovina. However, these studies used different referent samples. In order to contribute to the development of this database we decided to analyze 23 Y-STR loci, which included 11 additional loci compared to the previous number of Y-STRs, among which there were 6 new loci incorporated for the first time in the Y-STR multiplex kit (DYS576, DYS481, DYS549, DYS533, DYS570, DYS643).

## Materials and methods

### Sampling and extraction

This study was conducted on a population sample of 100 unrelated men from Bosnia and Herzegovina during 2012. The reference sample approximately proportionaly included the three main ethnic groups in Bosnia and Herzegovina: Bosnian Muslims (45%), Bosnian Serbs (34%), and Bosnian Croats (21%), with the M/F ratio of 0.97. The tested individuals were voluntary participants and gave the informed consent. Sample collection using buccal swabs was done in all geographical regions in Bosnia and Herzegovina. Genomic DNA was extracted from the buccal swabs using the salting out method (9), as well as Qiagen DNeasyTM Tissue Kit (10) (Qiagen, GmbH, Hilden, Germany).

### Genotyping

Polymerase chain reaction (PCR) was performed using the PowerPlex^®^ Y23 System (Promega Corporation) according to the manufacturer’s recommendations (11), which includes the loci DYS19, DYS385a/b, DYS389I/II, DYS390, DYS391, DYS392, DYS393, DYS437, DYS438, DYS439, DYS448, DYS456, DYS458, DYS481,DYS533,DYS549, DYS570, DYS576, DYS635, DYS643, and Y-GATA-H4. The PCR amplifications were carried out in PE GeneAmp PCR System Thermal Cycler (ABI, Foster City, CA, USA) according to the manufacturer’s recommendations. The 23 Y-chromosomal STR markers were typed using the ABI 310 Genetic Analyser (ABI, Foster City, CA, USA).

### Statistical analysis

Haplotype and allele frequencies were estimated by gene counting. Gene and haplotype diversities were calculated according to Nei (13) using the Arlequin software, V. 3.5 (12,13).

## Results

A total of 98 unique haplotypes were detected, and 1 (ID1) appeared two times (Supplementary Table)(Supplementary Table 1). The most polymorphic locus was DYS418, with 14 detected alleles (Table 1). This locus is one of 6 new loci added to the PowerPlex^®^ Y23 kit, which confirms the importance of increasing the number of Y STR loci included in forensic analysis. Furthermore, at the locus DYS418, we detected allele 33, which was not incorporated in the allelic ladder provided by the PowerPlex^®^ Y23 kit. The least polymorphic loci in our study were DYS389I, DYS391, DYS437, and DYS393.

**Table 1:** Allele frequency distribution and average gene diversity for the PowerPlex® Y23 System in a population sample from Bosnia and Herzegovina

Locus	Allele	Frequency	Locus	Allele	Frequency	Locus	Allele	Frequency	Locus	Allele	Frequency
DYS576	11		DYS389 I	9		DYS448	14		DYS389 II	24	
	12			10			15			25	
	13			11			16			26	
	14			12	0.110		17			27	
	15			**13**	**0.700**		18	0.040		28	0.050
	16	0.060		14	0.190		19	0.440		29	0.190
	17	0.280		15			**20**	**0.460**		30	0.330
	**18**	**0.390**		16			21	0.060		**31**	**0.340**
	19	0.170		17			22			32	0.080
	20	0.070					23			33	0.010
	21	0.030					24			34	
	22									35	
	23										
DYS19	9		DYS391	5		DYS481	17		DYS549	7	
	10			6			18			8	
	11			7			19			9	
	12	0.020		8			20	0.010		10	
	13	0.160		9	0.040		21	0.030		**11**	**0.460**
	14	0.150		10	0.440		22	0.160		12	0.420
	15	0.240		**11**	**0.520**		23	0.180		13	0.070
	**16**	**0.370**		12			24	0.070		14	0.050
	17	0.060		13			25	0.050		15	
	18			14			26	0.020		16	
	19			15			27	0.040		17	
				16			28	0.020			
							29	0.040			
							30	0.160			
							**31**	**0.200**			
							32	0.010			
							33	0.010			
DYS533	7		DYS438	6		DYS437	11		DYS570	10	
	8			7			12			11	
	9	0.010		8			13			12	
	10	0.010		9	0.070		14	0.440		13	
	11	0.160		**10**	**0.710**		**15**	**0.510**		14	
	**12**	**0.610**		11	0.180		16	0.050		15	
	13	0.210		12	0.040		17			16	
	14			13			18			17	0.110
	15			14						**18**	**0.430**
	16			15						19	0.310
	17			16						20	0.090
										21	0.050
										22	0.010
										23	
										24	
										25	
DYS635	15		DYS390	17		DYS439	6		DYS392	4	
	16			18			7			5	
	17			19			8			6	
	18			20			9	0.030		7	
	19			21			10	0.100		8	
	20	0.060		22	0.050		11	0.180		9	
	21	0.170		23	0.070		**12**	**0.430**		10	
	22	0.300		**24**	**0.610**		13	0.220		**11**	**0.880**
	**23**	**0.410**		24.3	0.050		14	0.040		12	0.020
	24	0.060		25	0.220		15			13	0.040
	25			26			16			14	0.050
	26			27			17			15	
	27			28						16	0.010
	28			29						17	
										18	
										19	
										20	
DYS643	6		DYS393	7		DYS458	10		DYS385a/b	7	
	7			8			11			8	
	8	0.010		9			12			9	
	9	0.080		10			13	0.010		10	
	**10**	**0.620**		11			14	0.030		11	0.130
	11	0.090		12	0.080		15	0.230		12	0.020
	12	0.180		**13**	**0.840**		16	0.160		13	0.070
	13	0.020		14	0.080		**17**	**0.300**		**14**	**0.310**
	14			15			17.2	0.010		15	0.220
	15			16			18	0.220		16	0.140
	16			17			19	0.030		17	0.035
	17			18			20			18	0.045
							21	0.010		19	0.030
							22			20	
							23			21	
							24			22	
										23	
										24	
										25	
										26	
										27	
										28	
DYS456	11		YGATAH4	8							
	12			9							
	13	0.010		10	0.010						
	14	0.120		**11**	**0.540**						
	**15**	**0.500**		12	0.370						
	16	0.230		13	0.050						
	17	0.110		14							
	18	0.020		15	0.030						
	19	0.010		16							
	20			17							
	21			18							
	22										
	23										

*Average gene diversity per locus: 0.619166 ± 0.309990. Major allele frequencies per locus are in bold.

## Discussion

A previous Y-STR study (4) was conducted on a different reference sample comprising 100 men from Bosnia and Herzegovina and including 12 loci. This study showed 69 unique haplotypes, 7 appeared twice, 4 appeared three times, and 1 appeared five times. Our study was done on the same number of reference samples from Bosnia and Herzegovina, but included 11 additional loci. The results showed 98 unique haplotypes and only one repetition. This indicates that by increasing the number of STR loci, the number of unique haplotypes increases and the number of repetitions decreases.

Furthermore, in the study of Y-STR diversity in Sarajevo region (14), which analyzed 12 loci using the PowerPlex^®^ Y kit, the most polymorphic loci were DYS385a and DYS385b. The least polymorphic loci were DYS 391, DYS389I, and DYS437. In our study, the least polymorphic loci were DYS 391, DYS389I, and DYS437, which confirms the previous results (4,14), but includes the DYS393 locus in the group of the least polymorphic loci. A quality control check was performed using the proficiency testing of the Y-STR Haplotyping Quality Assurance Exercise 2012 (15).

This study showed that increasing the number of highly polymorphic Y-STR markers, to include those tested in our analysis, leads to a reduction of repeating haplotypes, which is very important in the application of forensic DNA analysis.
