# Supplementary material for: Haplotype data for 23 Y-chromosome markers in a reference sample from Bosnia and Herzegovina
Source: Croat Med J. 2013 Jun;54(3):286–90. doi: 10.3325/cmj.2013.54.286 (PMC3692337; doi:10.3325/cmj.2013.54.286)
Supplement: Supplementary Table 1 [file CroatMedJ_54_s001.pdf]

Table 1. Y-short tandem repeat haplotypes in 100 unrelated men from Bosnia and Herzegovina*

Haplotype ID	DYS576	DYS389I	DYS448	DYS389 II	DYS19	DYS391	DYS481	DYS549	DYS533	DYS438	DYS437	DYS570	DYS635	DYS390	DYS439	DYS392	DYS643	DYS393	DYS458	DYS385a	DYS385b	DYS456	YGATAH4	n
ID1	18	13	20	30	17	11	23	12	12	11	14	19	23	25	11	11	9	13	15	11	13	16	15	2
ID2	18	13	19	31	16	11	30	11	12	10	15	19	23	24	12	11	10	13	18	14	15	15	11	1
ID3	17	13	19	31	15	11	29	11	12	11	15	18	23	24	12	11	10	13	16	15	15	15	11	1
ID4	20	13	19	31	17	11	31	11	12	10	15	19	23	24	13	11	10	13	17	14	15	15	11	1
ID5	17	14	20	29	14	10	24	12	12	10	14	19	21	24,3	12	14	11	14	18	11	16	14	12	1
ID6	17	14	20	29	14	10	24	12	13	10	14	18	21	24,3	13	14	11	14	19	11	16	14	12	1
ID7	18	12	20	29	13	10	26	12	11	10	14	17	22	24	12	11	9	13	17,2	14	19	15	10	1
ID8	18	13	19	31	16	11	31	11	12	10	15	18	23	24	12	11	10	13	17	14	15	15	11	1
ID9	18	13	19	30	12	10	22	13	12	10	14	19	21	24	9	11	12	13	17	16	17	16	12	1
ID10	19	13	19	31	15	11	31	11	12	10	15	18	22	24	13	11	10	13	18	14	16	15	11	1
ID11	18	13	19	30	16	11	30	11	12	10	15	18	23	24	12	11	10	13	15	14	15	15	11	1
ID12	17	13	20	30	13	9	23	14	12	10	14	19	21	24	12	11	12	13	16	17	18	16	12	1
ID13	19	12	19	30	15	11	30	11	12	10	15	18	21	24	13	11	10	13	18	14	15	15	11	1
ID14	17	14	20	30	15	10	25	12	11	11	14	21	23	24	11	11	10	13	15	11	11	17	12	1
ID15	18	13	19	30	16	11	30	11	13	10	15	18	23	24	13	11	10	13	18	14	15	15	11	1
ID16	18	13	20	29	16	10	25	12	12	11	14	19	23	25	11	11	10	13	16	11	14	19	12	1
ID17	21	13	20	31	16	10	30	11	13	10	15	17	24	24	13	11	10	13	21	14	15	15	11	1
ID18	19	13	20	32	15	10	28	11	12	10	15	18	22	24	13	11	10	13	17	14	15	15	11	1
ID19	16	12	20	29	15	10	24	12	11	10	16	21	20	22	11	11	12	13	14	12	14	15	11	1
ID20	18	14	19	32	15	11	30	11	12	10	15	18	24	24	13	11	11	13	16	14	15	15	11	1
ID21	19	13	19	31	16	11	31	11	12	10	15	19	23	24	14	11	10	13	18	14	15	15	11	1
ID22	17	14	18	33	16	11	30	11	13	10	15	18	23	24	12	11	10	13	18	14	15	15	11	1
ID23	17	14	20	31	13	10	22	12	11	10	14	19	22	25	12	11	12	13	15	16	18	16	12	1
ID24	19	13	19	31	15	11	31	11	12	10	15	18	22	24	13	11	10	13	19	14	15	16	11	1
ID25	19	14	20	30	13	9	27	11	11	10	14	22	21	24	10	11	12	13	18	13	14	16	12	1
ID26	18	13	20	30	13	10	22	12	12	10	14	20	23	25	11	11	12	13	15	16	18	17	12	1
ID27	18	13	19	31	16	11	31	11	13	10	15	18	23	24	13	11	10	13	17	14	15	15	12	1
ID28	18	13	18	31	16	10	30	11	13	10	15	18	22	24	13	11	10	13	17	14	15	16	11	1
ID29	19	13	19	32	16	11	31	12	13	10	15	19	22	24	13	11	10	13	17	14	15	15	11	1
ID30	18	13	19	31	16	10	30	11	13	10	15	17	22	24	12	11	10	13	17	13	15	15	11	1

ID31	18	13	19	31	16	11	27	11	12	10	15	19	23	24	13	11	9	13	18	14	15	16	11	1
ID32	18	12	19	30	16	10	31	11	12	10	15	19	23	24	12	11	10	13	17	14	16	15	11	1
ID33	18	13	20	31	16	11	23	12	12	11	14	19	24	25	10	11	10	13	15	11	14	15	13	1
ID34	20	13	19	30	16	11	28	11	12	10	15	18	23	25	12	11	10	13	18	15	16	15	11	1
ID35	19	13	18	31	15	11	31	11	11	10	15	18	24	25	13	11	10	13	18	14	16	15	11	1
ID36	17	13	20	30	13	10	22	12	12	10	14	19	23	25	11	11	12	13	15	16	18	17	12	1
ID37	18	13	19	30	16	11	31	11	12	10	15	18	22	24	13	11	10	13	18	14	15	15	11	1
ID38	19	13	19	31	15	11	31	11	12	10	15	18	22	24	12	11	10	13	17	14	16	15	11	1
ID39	18	13	20	30	16	11	31	11	12	10	15	19	22	24	12	11	10	13	17	14	15	15	11	1
ID40	17	13	19	29	14	11	23	14	12	12	15	18	23	24	12	13	10	13	15	11	11	15	12	1
ID41	19	13	20	30	17	10	23	13	12	10	14	18	21	25	10	11	10	13	15	11	14	16	13	1
ID42	17	13	20	30	13	10	23	13	12	10	14	19	22	24	12	11	12	13	15	16	18	16	11	1
ID43	17	12	20	28	14	10	25	12	11	10	16	21	22	23	12	11	11	13	15	13	14	14	12	1
ID44	17	12	19	28	15	10	23	12	11	9	14	17	21	23	12	12	9	12	16	15	19	14	12	1
ID45	19	13	19	31	16	11	31	11	13	10	15	18	22	24	12	11	11	13	17	13	15	14	11	1
ID46	18	14	18	31	15	11	31	11	13	10	15	18	23	23	12	11	10	13	17	14	15	15	11	1
ID47	17	14	19	29	14	10	24	12	12	10	14	18	21	24,3	12	14	11	14	19	11	16	14	12	1
ID48	17	13	21	32	16	11	29	11	12	10	15	19	23	24	13	11	10	13	17	14	14	15	11	1
ID49	20	13	20	30	13	10	22	12	12	10	14	20	21	25	12	11	12	13	15	19	19	15	12	1
ID50	18	13	19	30	13	10	22	12	12	10	14	19	21	24	9	11	12	13	17	16	18	16	12	1
ID51	17	13	19	30	15	11	33	11	13	10	15	20	23	25	12	11	10	13	18	14	15	15	11	1
ID52	17	14	20	29	14	10	23	12	12	10	14	18	21	24,3	12	14	11	14	18	11	16	14	12	1
ID53	18	13	20	31	15	11	30	11	13	10	15	18	22	24	14	11	10	13	18	14	15	15	11	1
ID54	19	13	19	30	14	11	30	11	13	10	15	18	22	24	13	11	10	13	17	14	15	15	12	1
ID55	17	13	20	30	13	10	22	12	12	10	14	20	20	24	11	11	12	13	15	16	19	17	12	1
ID56	18	14	20	30	15	10	26	12	12	11	15	20	23	25	11	11	10	13	18	12	14	17	12	1
ID57	19	14	20	31	12	10	22	12	12	10	14	20	22	24	11	11	12	13	17	16	18	16	11	1
ID58	17	13	19	31	13	11	29	11	12	10	15	18	23	24	14	11	10	13	17	14	15	15	11	1
ID59	19	14	20	32	13	10	23	12	12	10	14	18	22	24	12	11	12	13	17	16	17	16	12	1
ID60	16	12	19	29	15	10	23	13	10	9	16	20	21	24	14	11	9	12	16	14	17	13	11	1
ID61	18	13	21	31	14	10	22	12	11	9	15	18	22	23	12	11	10	13	14	13	17	14	11	1
ID62	17	12	20	28	16	11	23	12	12	11	14	21	23	24	10	11	10	13	15	11	15	15	13	1
ID63	21	13	20	30	15	10	22	12	12	11	14	17	23	25	10	11	10	13	15	11	14	16	12	1
ID64	18	13	19	29	14	11	22	13	12	12	15	17	23	24	12	13	10	13	16	11	11	15	12	1
ID65	18	13	20	30	13	10	22	12	12	10	14	19	20	24	11	11	12	13	15	16	19	16	12	1

ID66	17	13	21	32	16	10	22	12	11	9	14	17	20	22	12	11	8	12	15	13	15	15	12	1
ID67	18	13	20	31	16	11	31	12	12	10	15	18	22	24	12	11	10	13	17	14	15	15	11	1
ID68	16	12	20	28	14	10	27	12	11	10	15	19	22	23	11	11	13	13	15	13	14	14	11	1
ID69	16	13	21	31	13	10	22	12	12	10	14	21	22	24	12	11	13	13	16	16	18	18	12	1
ID70	17	12	21	29	15	10	21	11	9	10	16	18	20	22	12	11	12	13	16	14	14	15	11	1
ID71	17	13	19	31	16	11	31	11	13	10	15	19	21	24	12	11	10	13	17	14	16	15	11	1
ID72	16	13	21	29	15	9	23	12	13	9	14	19	21	24	12	11	10	12	14	13	16	16	12	1
ID73	21	13	20	31	13	10	23	13	12	10	14	18	22	24	12	11	12	13	17	16	17	16	12	1
ID74	20	13	20	32	16	11	30	11	11	10	15	18	22	24	13	11	10	13	16	14	15	15	11	1
ID75	18	14	19	32	16	11	29	11	13	10	15	18	23	24	13	11	10	13	18	14	15	18	11	1
ID76	20	13	20	30	16	10	21	12	13	11	14	17	23	25	10	11	10	13	16	11	14	17	12	1
ID77	17	13	20	31	15	11	30	11	13	10	15	17	22	24	12	11	10	14	17	14	15	15	11	1
ID78	17	14	20	29	14	10	24	12	12	10	14	19	21	24,3	12	14	11	14	17	11	16	14	12	1
ID79	18	13	20	29	16	11	24	12	12	11	14	19	23	25	10	11	10	13	13	11	14	16	12	1
ID80	17	13	20	29	16	10	25	12	11	11	14	19	23	25	11	11	10	13	16	11	14	17	12	1
ID81	17	12	20	28	14	10	27	13	11	10	16	18	21	22	11	11	12	13	16	14	14	15	11	1
ID82	18	13	19	31	16	11	30	11	12	11	15	18	23	24	12	11	10	13	17	14	15	14	11	1
ID83	17	13	19	30	15	11	31	11	12	10	15	18	23	24	13	11	10	13	17	14	15	15	11	1
ID84	18	13	19	30	15	11	31	11	13	10	15	18	23	24	13	11	10	13	18	14	15	15	11	1
ID85	18	13	19	31	16	11	31	11	13	10	15	18	22	24	12	11	10	13	17	14	15	15	12	1
ID86	18	13	20	30	17	11	23	12	12	11	14	19	23	25	11	11	9	14	15	11	13	16	15	1
ID87	17	13	19	30	17	11	31	11	12	10	15	18	24	24	13	11	10	13	18	14	16	15	11	1
ID88	18	14	20	31	13	10	22	12	12	10	14	19	23	24	11	11	12	13	15	16	18	16	12	1
ID89	19	14	19	29	14	10	23	14	12	9	14	17	20	22	11	11	9	12	18	15	17	15	12	1
ID90	16	14	19	30	14	11	20	12	11	11	14	20	22	23	9	16	11	13	16	11	13	14	13	1
ID91	19	13	19	29	15	11	22	14	12	12	15	18	23	24	12	13	10	12	18	12	16	17	11	1
ID92	18	13	20	29	16	10	25	12	12	11	14	19	23	25	11	11	10	13	16	11	14	17	11	1
ID93	18	13	19	31	16	11	30	11	13	10	14	18	23	25	12	11	10	13	17	14	16	15	11	1
ID94	18	13	19	31	16	11	30	11	12	10	15	19	22	24	12	11	10	13	17	14	15	15	11	1
ID95	20	14	20	31	16	10	21	12	12	11	14	18	23	25	10	11	10	14	15	11	13	17	12	1
ID96	20	13	20	30	16	11	23	12	12	11	14	19	24	25	10	11	10	13	16	11	14	16	13	1
ID97	18	13	20	30	14	9	24	11	11	9	15	18	22	23	10	12	10	12	15	13	15	17	11	1
ID98	19	13	19	31	16	11	32	11	12	10	15	20	22	24	12	11	10	13	17	14	15	15	11	1
ID99	18	13	19	29	15	11	23	14	12	12	14	17	23	24	12	13	10	12	18	12	15	16	11	1

*Haplotype diversity (h), 0.9998 ±0.0015.
